# Antioxidant Potential of the Extracts, Fractions and Oils Derived from Oilseeds

**DOI:** 10.3390/antiox2040246

**Published:** 2013-10-15

**Authors:** Shagufta Ishtiaque, Nasir Khan, Muhammad A. Siddiqui, Rahmanullah Siddiqi, Shahina Naz

**Affiliations:** 1 Department of Chemical Engineering, University of Karachi, Karachi 75270, Pakistan; E-Mail: shagiaslam@yahoo.com; 2 Department of Food Science & Technology, University of Karachi, Karachi 75270, Pakistan; E-Mails: nasirkhanfst@hotmail.com (N.K.); asadg@hotmail.com (M.A.S.); rahman_siddiqi@yahoo.co.uk (R.S.)

**Keywords:** antioxidant activity, polyphenols, ajwain, fenugreek, poppy, mustard, seed oil

## Abstract

The polyphenolic extracts and oils were obtained from ajwain, mustard, fenugreek and poppy seeds*.* The extracts were partitioned into acidic and neutral polyphenolic fractions and following estimation of total phenolics in the crude extract, acidic and neutral fractions and oil, all were analyzed for their DPPH (2,2-diphenyl-1-picrylhydrazyl) scavenging potential, ferric reducing ability and chelating power. The highest amount of polyphenols was found in ajwain (8330 ± 107), then in mustard seeds (2844 ± 56.00) and in fenugreek (1130 ± 29.00), and least in poppy seeds (937 ± 18.52). The higher amounts of polyphenols were estimated in neutral fraction compared to acidic (*p* < 0.05). % Inhibition of DPPH by the crude extract and fractions of all oilseeds was quite significant, being higher for acidic than neutral. The highest % DPPH inhibition was shown by ajwain extract than mustard > fenugreek and least by poppy seed extracts (*p* < 0.05). The reducing power and the chelating effect of the oilseeds followed the same order as DPPH, but higher % chelation was shown by neutral than acidic fraction (*p* < 0.05). Though low in polyphenols, the oil fractions were as strong antioxidants as the acidic one. Though oilseeds are used in very small quantity in food, they are potential sources of natural antioxidants and may replace synthetic ones.

## 1. Introduction

The foods containing antimutagenic, antibacterial, antiviral and anti-inflammatory phenolic compounds have increasingly been gaining importance since numerous studies have proved strong protective effects of these novel phytochemicals against many diseases [1,2,3,4]. People, due to increasing awareness and unbearable side effects, are now more reluctant to use conventional drugs. Most of the medicinal effects of the phenolic compounds are derived from their antioxidant potential [1,2,3,4,5,6,7,8,9], which enable to them to adsorb and neutralize free radicals, quench singlet and triplet oxygen, or decompose peroxides [10,11]. These reactive oxygen species produced in response to normal cellular metabolism, if not captured by antioxidants, may cause oxidative damage to biomolecules and disorders like cancer, diabetes, asthma, inflammatory, cardiovascular and neurodegenerative diseases, and premature aging [1,12,13].

Polyphenolic antioxidants do not only help combat with the reactive oxygen species but also minimize rancidity, reduce the formation of toxic oxidation products, maintain nutritional quality and extend shelf life when added to foods [14,15]. The antioxidant activity of several polyphenolic extracts and compounds derived from leaves, fruits, seeds, bark, roots and oilseeds has been extensively studied and documented [14,15].

Spices and/or oilseeds are an important category of food that has been used since long to enhance the taste and aroma of foods. Besides imparting characteristic flavor and color to foods, they also produce several medicinal effects and hence are used in several indigenous systems of medicines [16,17,18,19]. Few spices and oilseeds have been shown to impart several beneficial effects of which the antioxidant effect is most pronounced [20,21]. It has been found that spices have higher antioxidant activity as compared to fruits, cereals and nuts. The active components in spices- phthalides, polyacetylones, phenolic acids, flavonoids, coumarins and terpenes are reported as powerful antioxidants [22,23]. The spice oilseeds like mustard, fenugreek, poppy, black cumin and coriander have not only been found to be effective antioxidant *in vivo* and *vitro* to deal with oxidation stresses but also quite active in stabilizing the edible oils and fatty food against rancidity and oxidative deterioration [24].

The oilseeds *Trachyspermum ammi* (ajwain), *Brassica alba* (mustard/rai), *Trigonellafoenum graecum* **(**Feenugreek) and *Papaver somniferum* (poppy seeds/khashkhash) were extracted for polyphenols and oil using Bligh and Dyer method. Polyphenolic extracts were then partitioned into neutral and acidic polyphenolic fractions. The polyphenolic and oil fractions were then compared for their antioxidant potential to seek the possibility of using them either in medicinal preparations or as preservatives for frying edible oil and high fatty foods.

## 2. Experimental Section

### 2.1. Materials

The oilseeds were purchased from the local market, washed to remove extraneous matter, dried in open air turning up and down time to time and then stored in portions in polyethylene bags at room temperature. Lichrolut RP-18 SPE (solid phase extraction) cartridges were supplied by Merck and all other reagents and chemicals were of analytical grade supplied either by Merck or BDH.

### 2.2. Extraction of Oil and Polyphenols from Oilseeds

One kg of each oilseed was ground to coarse size using an electric grinder (Braun, Frankfurt Germany). In order to avoid losses of heat sensitive antioxidants, and to make the process more feasible, the oil was extracted with chloroform-methanol mixture by Bligh and Dyer method [25] with the intention of simultaneously recovering polyphenols from methanol-water mixture. 

One kg of the ground sample was added to 3 L of CHCl_3_/MeOH (1:2, v/v) and homogenized well. Subsequent to this, 1 L of CHCl_3_ was added, homogenized, 1 L of doubled distilled water was added, homogenized again and then finally centrifuged at 1000 rpm for 5 min at room temperature to give a two-phase system (MeOH-water, top) and CHCl_3_ (bottom). 95% of the bottom phase were recovered with the assistance of separating funnel while the remaining 5% were collected following addition of 100–200 mL of CHCl_3_, vigorously shaking and collecting from bottom. Oil fraction was recovered from the chloroform phase by evaporating CHCl_3_ on rotary evaporator at 40 °C while for polyphenols; methanolic phase was concentrated to remove alcohol and resolubilized in water. To retain the stability of oil portion, moisture was removed by adding anhydrous sodium sulfate. The clear dry oil was then collected by decantation and stored at 4 °C till further study. Yield of oil in percent and total extractable solids from aqueous methanol were calculated for each oilseed.

### 2.3. Fractionation of Crude Polyphenolic Extracts into Acidic and Neutral Fractions

Polyphenolic extract of each oilseed was fractionated into acidic and neutral polyphenols by using the procedure of Oszmianski and Lee [26]. Two polypropylene columns (82 mm × 20 mm) filled with 5 g of Lichroprep RP 18 column (25–40 µm) were used for this separation. One of the columns was preconditioned with methanol (10 mL) and then water (10 mL); subsequently, the polyphenolic extracts (neutralized to pH 7.0 with 5 N NaOH) was passed through the column to absorb the neutral compounds and the effluent was collected as acidic fraction. The effluent (pH adjusted to 2.0 with 1 N NaOH) was passed through the second column preconditioned with methanol (10 mL) and 0.01 N HCl (10 mL). Both neutral and acidic polyphenolics were then eluted from their respective columns with 10 mL absolute methanol. Methanol was removed from both and the residues were resolubilized in water and stored at 4 °C till further analyses.

### 2.4. Determination of Total Phenolics

The total phenolics in the crude polyphenolic extracts, acidic, neutral and oil fractions were determined by the method of Jayaprakasha *et al*. [27], however prior to the determination of total phenolics in the oil fraction, polyphenols were extracted from the oil by the method of Ramadan *et al*. [28]. The oil fraction was dissolved in *n*-hexane (1:2 w/v). The dissolved oil was mixed with MeOH/water (80:20, v/v) in the ratio of 1:5 (w/v), vortexed for 2–3 min and then centrifuged at 3000 rpm for 10 min. The aqueous MeOH was separated from lipid phase, concentrated and set aside. The lipid residue was redissolved in MeOH/water (80:20, v/v) and the extraction was repeated. The aqueous MeOH extracts so obtained were combined, redissolved in acetonitrile and washed with *n*-hexane. The polyphenols recovered in acetonitrile were concentrated under vacuum at 30 °C, dissolved in methanol and then analyzed for total phenols. Crude extracts were also analyzed for total flavonoids by the method of Zhishen *et al*. [29].

### 2.5. Antioxidant Activity of the Extract, Fractions and Oil

The antioxidant potential of the polyphenolics extract, fractions and oil was determined by three different methods: 2,2-diphenyl-1-picrylhydrazyl (DPPH) assay, reducing power assay and Fe(II) chelating activity. Butylated hydroxyl anisole (BHA) was used as a positive control in these assays.

#### 2.5.1. Radical Scavenging Activity by DPPH Assay

The DPPH scavenging activity was evaluated according to Negi *et al*. [30] with little modification. 0.1 mL of the crude extract, acidic and neutral fraction in varying concentration (50–250 µg/100 µL in methanol) was treated with methanolic solution of DPPH (1.4 mL; 0.2 mM) and 1.5 mL of distilled water, mixed thoroughly in vortex and the mixture was placed in the dark for 30 min. The decrease in absorbance was measured at 515 nm against a blank using a spectrophotometer. The DPPH activity of the oil obtained from each oilseed was determined by reduction of DPPH in toluene [28]. The oil in concentration of 100–250 µg/100 µL (in toluene) was treated with toluenic solution of DPPH (1.5 mL; 0.2 mM) and the decrease in absorbance was recorded as above. Percent DPPH inhibition was calculated as Lee *et al*., (2002) [31].

#### 2.5.2. Ferric Reducing Power Assay

Ferric reducing power was determined by the method of Jayaprakasha *et al*. [32]. To the extract and fractions (50–250 µg/mL in methanol), 2.5 mL of phosphate buffer (0.2 M, pH 6.6) and 2.5 mL of potassium ferricyanide (1%) were mixed and incubated for 20 min at 50 °C. 2.5 mL of trichloroacetic acid (10%) was then added and centrifuged for 10 min at 5000 rpm. To 2.5 mL of the top layer, 2.5 mL dd water and 0.5 mL FeCl_3_ (0.1%) were added and the absorbance was noted at 700 nm. The reducing power of the samples was determined by observing increase in absorbance.

#### 2.5.3. Chelating Effect

Chelating activity (Fe^2+^) was measured by 2,2ʹ-bipyridyl assay [33]. To 0.25 mL of FeSO_4_ solution (1 mM) was added 0.25 mL of extract or fraction (50–250 µg/mL in ethanol), 1 mL of Tris-HCl buffer (pH 7.4), 1 mL 2,2ʹ-bipyridyl solution (0.1% in 0.2 M HCl) and 2.5 mL of ethanol. The absorbance was measured at 522 nm and used to evaluate Fe^2+^ chelating activity using Na_2_EDTA as a standard.

#### 2.5.4. Statistical Analysis

For the statistical analysis, three replicates of each sample were used and the data were expressed as mean ± S.D. Pearson correlation test was conducted to determine the correlation between total phenolics and antioxidant activity. To identify significant differences among the mean values (at *p* < 0.05) analysis of variance (ANOVA) and Tukey’s tests were performed.

## 3. Results and Discussion

The amounts of total extractable solids in mg/g from aqueous methanolic phase of all the oilseeds are given in the Table 1. The highest amounts of solids were extracted from ajwain while least from fenugreek. The highest polyphenol contents were found in ajwain, then in mustard > in fenugreek and least in poppy seeds. In all oilseeds the higher amount of polyphenols were estimated in neutral fraction compared to acidic (*p* < 0.05) (Table 2).

**Table 1:** Total extractable solids, total phenolics, flavonoids and % oil yield of the oilseeds.

Sample oilseed	Total extractable solids mg/g sample	Total phenolics mg GAE/100 g extract	Total flavonoids mg QE/100 g extract	% oil yield
Ajwain	240.5 ± 29.0	8330.5 ± 107.0	5343.5 ± 60.3	4.0 ± 0.1
Mustard	170.4 ± 31.0	4144.6 ± 56.0	987.3 ± 32.7	21.0 ± 2.2
Fenugreek	130.3 ± 21.0	3130.5 ± 29.0	580.5 ± 38.0	6.9 ± 1.1
Poppy	156.7 ± 15.5	1937.7 ± 28.5	676.3 ± 29.3	16.2 ± 2.1

Results are expressed as mean ± standard deviation (*n* = 3, *p* < 0.05). GAE and QE stands for gallic acid and quercetin equivalents respectively.

**Table 2:** Total phenolics of the crude extract, netural, acidic and oil fraction.

Oilseed	Total phenolics (mg GAE/100 g)			
	Crude extract	Neutral fraction	Acidic fraction	Oil fraction
Ajwain	8330.5 ± 107.0	4070.5 ± 74.5	2900.2 ± 25.6	1923.4 ± 29.0
Mustard seeds	4144.6 ± 56.0	2100.6 ± 26.7	800.5 ± 17.9	360.6 ± 13.0
Fenugreek	3130.5 ± 29.0	1320.3 ± 18.9	430.3 ± 12.0	100.6 ± 9.0
Poppy seeds	1937.7 ± 28.5	870.5 ± 23.0	190.2 ± 17.0	48.5 ± 3.0

Results are expressed as mean ± standard deviation (*n* = 3, *p* < 0.05). GAE stands for gallic acid equivalents.

% Inhibition of DPPH by the crude extract and fractions of all oilseeds was quite significant and concentration dependent (Table 3). Among the oilseeds, the highest % DPPH inhibition was shown by ajwain extract then mustard > fenugreek and least by poppy seed extracts (*p* < 0.05) (Table 3).

**Figure 1:**
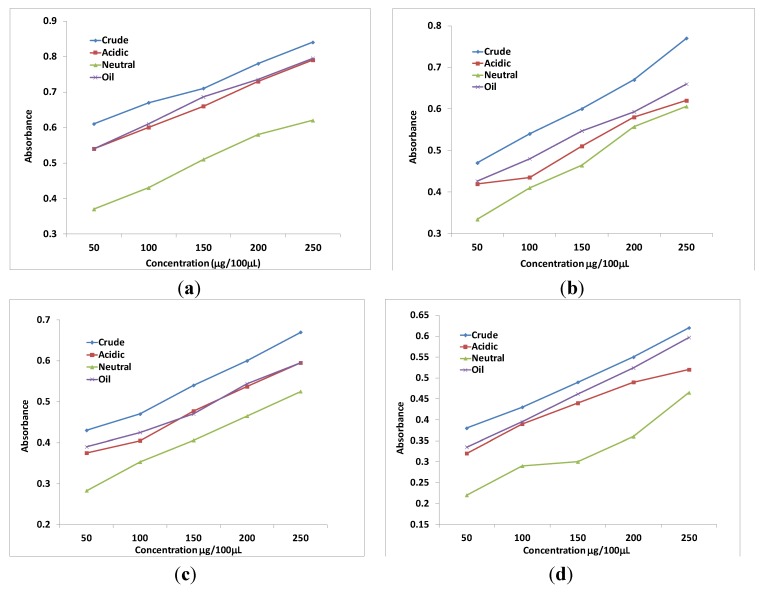
Reducing power of crude extract, acidic and neutral fraction and oil from (**a**) Ajwain seeds, (**b**) mustard, (**c**) fenugreek and (**d**) poppy seeds. Results are expressed as mean ± standard deviation (*n* = 3), all significantly different (*p* < 0.05) except difference between acidic fraction and oil.

Like % DPPH inhibition, the highest reducing power was shown by ajwain extract and fractions. The reducing power shown by ajwain was comparable to the reducing power shown by BHA at all concentrations. Rest of the fractions showed the same order of reducing power as in DPPH (Figure 1). The chelating effect of all the extracts and fractions were dependent upon concentration and followed the same order *i.e.*, ajwain > mustard > fenugreek > poppy seeds but unlike DPPH, neutral fraction > acidic fraction (*p* < 0.05) (Table 4).

**Table 3:** DPPH scavenging activity of the crude extract, acidic, neutral and oil fraction.

Sample (µg/100 µL)	% Inhibition of DPPH			
	Crude extract ^a^	Acidic fraction ^b^	Neutral fraction ^c^	Oil fraction ^d^
Ajwain				
50	66.3 ± 1.5	60.4 ± 2.6	51.3 ± 2.4	60.3 ± 1.4
100	74.5 ± 1.0	69.5 ± 2.9	61.6 ± 3.0	67.6 ± 2.0
150	83.6 ± 2.3	76.5 ± 1.6	68.6 ± 2.1	77.5 ± 2.3
200	90.6 ± 2.8	85.4 ± 1.9	77.5 ± 2.4	83.0 ± 1.8
250	95.7 ± 3.0	91.6 ± 2.9	83.4 ± 1.8	88.0 ± 2.1
Mustard seeds				
50	57.0 ± 1.5	45.2 ± 1.2	37.1 ± 0.6	51.0 ± 1.5
100	59.2 ± 0.8	52.6 ± 1.7	44.2 * ±* 1.5	56.2 ± 0.9
150	66.5 ± 2.5	61.8 ± 2.1	54.4* ±* 2.1	60.5 ± 2.6
200	73.5 ± 2.1	68.7 ± 1.5	59.6* ±* 3.0	67.5 ± 2.1
250	79.4 ± 1.5	74.5 ± 0.9	65.8 ± 2.9	73.4 ± 1.6
Fenugreek				
50	50.4 ± 0.5	43.2 ± 2.3	35.1 ± 2.3	43.6 ± 1.5
100	55.3 ± 1.6	49.5 ± 2.5	40.6 ± 2.2	49.7 ± 2.6
150	62.0 ± 2.1	56.5 ± 1.8	47.7 ± 2.1	54.2 ± 1.1
200	67.0 ± 1.9	61.4 ± 1.9	55.8 ± 1.9	59.5 ± 1.8
250	73.0 ± 1.8	67.2 ± 2.1	60.7 ± 1.1	65.5 ± 2.8
Poppy seeds				
50	44.0 ± 1.0	38.5 ± 0.9	29.0 ± 1.5	37.2 ± 1.0
100	53.0 ± 1.5	43.5 ± 1.3	35.0 ± 3.0	44.7 ± 1.5
150	59.0 ± 2.3	49.5 ± 1.5	40.0 ± 2.8	49.5 ± 2.3
200	63.0 ± 1.8	55.0 ± 1.9	46.0 ± 2.7	54.0 ± 1.8
250	66.5 ± 0.6	62.0 ± 3.0	55.5 ± 1.6	60.5 ± 0.6

Results are expressed as mean ± standard deviation (*n* = 3). Values with in a column and with in a row were all significantly different (*p* < 0.05) except between column b and d.

**Table 4:** % Fe^2+^ chelating activity of the crude extract, acidic and neutral fraction and oil.

Sample (µg/100 µL)	% Chelating effect			
	Crude extract	Acidic fraction	Neutral fraction	Oil fraction
Ajwain				
50	49.3 ± 2.4	27.0 ± 2.7	36.6 ± 2.1	19.0 ± 2.7
100	55.0 ± 3.0	33.5 ± 1.2	42.3 ± 3.0	23.5 ± 1.2
150	60.2 ± 3.1	40.5 ± 1.7	44.2 ± 3.0	30.5 ± 1.7
200	66.7 ± 3.1	46.5 ± 1.9	49.0 ± 2.0	36.5 ± 1.9
250	77.7 ± 1.7	51.0 ± 1.1	57.5 ± 1.0	41.0 ± 1.1
Mustard seeds				
50	35.9 ± 2.1	22.5 ± 1.2	31.8 ± 2.1	12.5 ± 1.2
100	40.6 ± 3.0	28.8 ± 2.8	37.6 ± 3.0	18.8 ± 2.8
150	46.5 ± 2.8	32.5 ± 2.00	43.3 ± 2.8	22.5 ± 2.0
200	53.5 ± 1.7	39.5 ± 1.1	46.3 ± 1.7	29.5 ± 1.1
250	65.4 ± 2.1	46.5 ± 1.3	50.0 ± 2.1	35.5 ± 1.3
Fenugreek				
50	39.0 ± 2.1	18.4 ± 0.5	25.5 ± 2.5	-
100	44.0 ± 2.1	25.6 ± 1.7	30.5 ± 2.1	-
150	49.5 ± 1.5	27.6 ± 2.1	33.0 ± 1.5	17.6 ± 2.1
200	53.5 ± 1.8	33.3 ± 1.3	39.0 ± 2.0	23.3 ± 1.3
250	61.0 ± 1.5	41.0 ± 3.0	46.0 ± 1.6	30.0 ± 3.0
Poppy seeds				
50	33.0 ± 1.9	17 ± 0.8	22.0 ± 1.9	-
100	39.0 ± 1.9	21 ± 1.2	25.0 ± 1.9	-
150	41.0 ± 1.9	26 ± 2.1	29.0 ± 1.9	16 ± 2.1
200	49.6 ± 1.9	31 ± 2.9	32.6 ± 1.9	20 ± 2.9
250	56.4 ± 1.7	36 ± 3.1	39.4 ± 1.7	25 ± 3.1

Results are expressed as mean ± standard deviation (*n* = 3). Values with in a column and with in a row were all significantly different (*p* < 0.05).

% yield of the oil obtained from the oilseeds has been depicted in Table 1. Though least in % yield, the oil obtained from ajwain showed maximum radical scavenging, reducing power and % chelating activity. With respect to total phenolics and antioxidant potential in the oil fraction, the order was same as for the methanolic crude extract and fractions of the oilseeds.

% DPPH inhibition indicated that polyphenolics extracts of oilseeds have substantial potential to scavenge the free radicals. The higher % inhibition of DPPH by acidic fraction showed relatively higher efficiency of phenolic acids to scavenge the free radicals. Though the total phenols were higher in neutral fraction than acidic yet the activity of the acidic fraction was higher than neutral. This indicated that the antioxidant activity is not only based the total phenolics but also on the nature of the phenolic compounds [11,34]. The antioxidant effect of phenolic acids is associated to the number and position of hydroxyl groups in the molecule, the higher is the number of hydroxyl groups on the phenyl radical of an acid, the higher is the antioxidant potential [10]. The catechol (*o*-dihydroxy group) structures in the aromatic ring of the phenolic compounds like caffeic and gallic acids enable them to form intramolecular hydrogen bonds with the free radicals and enhancing high antioxidant activity of these molecules in the extracts predominantly containing these species [21]. According to Naczk and Shahidi [35], the predominant phenolic compounds of oilseeds belong to phenolic acids, coumarins, flavonoids and tannins. Thus the higher % DPPH scavenging activity of phenolic acid fractions of the oilseeds may be due to their concentration and partly the structural features ensuring maximum radical scavenging activity.

At a concentration of 100–250 µg/100 µL, the activity of ajwain (74%–95%) was comparable to BHA (78%–97%) which means that use of synthetic antioxidants like BHA, BHT could be avoided by replacing them with the natural ones that not only effectively scavenge the free radicals but also preclude the side effects like liver damage caused by these synthetic antioxidants. A low correlation (*R*^2^ = 0.25–0.36) was found between the total phenolics in crude extract, neutral and acidic fractions and the % DPPH activity of the oilseeds indicating that there are some other factors responsible for antioxidant activity in the oilseeds besides the polyphenols. Significant antioxidant activity of acetone extract of ajwain in linoleic acid system, owing to the presence of compounds like thymol (39.1%), has already been demonstrated by Singh *et al*. [23]. In a study based on the antioxidant activity of the aqueous extracts of 15 spices including mustard, fenugreek and poppy seeds [21], the same order of activity was found as in our study *i.e.*, mustard > fenugreek > poppy seeds however, compared to the EC_50_ values determined in our study (mustard < 50 µg/100 µL; fenugreek 50 µg/100 µL; poppy 50–100 µg/100 µL) the EC_50_ values reported by Chan *et al*. [21] were very high. This may be due to the difference in extracting solvent and method of extraction.

The higher the antioxidant potential, the higher is the conversion of Fe^3+^ in ferric chloride to Fe^2+^. Like % DPPH, there was no linear correlation between the total phenolics and reducing power confirming that besides phenolics, other compounds present in the extracts and fractions of oilseeds were responsible for their reducing power. Non linear correlation may also be noticed due to the fact that oxidation reduction potential does not necessarily correlate to antioxidant properties [36]. In addition, the reducing power of acidic fraction was higher than neutral (*p* < 0.05).

% Chelating effect shown by the oilseeds extracts and fractions demonstrate that they not only have the ability to combat oxidation reactions directly but can also decelerate the oxidation reactions by chelating prooxidant metals. Transition metals like copper, iron and nickel are prooxidant and thus promote oxidation by acting as catalyst. Therefore sequestering of these metals ions by antioxidants decrease their prooxidant potential by stabilizing their oxidized form [37]. The % chelation for the ajwain extract and fractions (27%–77%) were comparable to EDTA at all concentrations (30%–78%).

In general the oil fraction was found to be more powerful radical scavenger and reducing agent than the corresponding neutral fraction; however the chelating power of the oil fractions was least. Total polyphenols were very low in oil fraction as expected; however, the oil fraction was as active as the acidic one with respect to the antioxidative potential. This may be justified on the basis of explanation given by Ramadan *et al*. [28]. According to their findings the antioxidant activity of oils from oilseeds is not only related to total phenolics but also positively correlate to total polar lipids, differences in composition of polar lipids and unsaponifiables, and structural diversity of potential phenolics in the crude oil. In non-refined oils, phenols make up the part of the polar fraction of oils and contribute to their antioxidant potential according to their concentration [28].

## 4. Conclusions

The results of the study show that, though oilseeds are used in very small quantity in food, they are potential sources of antioxidants. The study also suggests that the natural antioxidants may preferably be used for food preservation and securing health effects, as they are equally good in strength to synthetic ones.
